# Children Infected With SARS-CoV-2 From Family Clusters

**DOI:** 10.3389/fped.2020.00386

**Published:** 2020-06-23

**Authors:** Dan Sun, Feng Zhu, Cheng Wang, Jing Wu, Jie Liu, Xue Chen, Zhisheng Liu, Zubo Wu, Xiaoxia Lu, Jiehui Ma, Hua Peng, Han Xiao

**Affiliations:** ^1^Department of Neurology, Tongji Medical College, Wuhan Children's Hospital, Huazhong University of Science and Technology, Wuhan, China; ^2^Clinic Center of Human Gene Research, Tongji Medical College, Union Hospital, Huazhong University of Science and Technology, Wuhan, China; ^3^Department of Cardiology, Tongji Medical College, Union Hospital, Huazhong University of Science and Technology, Wuhan, China; ^4^Department of Epidemiology and Biostatistics, School of Public Health, Tongji Medical College, Huazhong University of Science and Technology, Wuhan, China; ^5^Pediatric Department, Tongji Medical College, Union Hospital, Huazhong University of Science and Technology, Wuhan, China; ^6^Department of Respiratory, Tongji Medical College, Wuhan Children's Hospital, Huazhong University of Science and Technology, Wuhan, China; ^7^Institute of Maternal and Child Health, Tongji Medical College, Wuhan Children's Hospital, Huazhong University of Science and Technology, Wuhan, China

**Keywords:** SARS-CoV-2, children, family clusters, COVID-19, viral pneumonia

## Abstract

**Background:** The outbreak of severe acute respiratory syndrome coronavirus 2 (SARS-CoV-2) is ongoing globally. Limited data are available for children with SARS-CoV-2 infection.

**Methods:** A retrospective case study was conducted in one designated hospital for children with SARS-CoV-2 infection in Wuhan.

**Results:** Out of the 74 children with laboratory-confirmed SARS-CoV-2 infection, the median age was 5.8 years, with no notable variation based on gender. All of the children had had direct exposure to at least one family member with confirmed SARS-CoV-2 infection. The most common symptoms were cough in 41 (55.4%) and fever in 38 (51.4%). Typical CT patterns of viral pneumonia were exhibited in 40 (54.1%) children, including ground-glass opacity and interstitial abnormalities. However, 17 (23.0%) children were classified as asymptomatic carriers, with neither symptoms nor radiological findings. Also, 68 (91.9%) children recovered fully and showed negative results on RT-PCR assay by nasopharyngeal swabs during our observation period. In contrast to the negative result for nasopharyngeal swab, 34% of the anal swabs showed a continued positive result. The mean hospitalization days of the children discharged after full recovery was 10.0 days.

**Conclusion:** Within family clusters that had SARS-CoV-2 infection, children had mild or even asymptomatic illness. Although CT is highly sensitive, it should be avoided in follow-up of the disease in consideration of the radiological hazards and limited clinical benefits for mild illness in children. Furthermore, it is advocated that both nasopharyngeal and anal swabs should be confirmed negative for viral load prior to declaring full recovery so as to avoid oral-fecal transmission. Asymptomatic children with family clusters are potentially a little-known source of COVID-19. This therefore warrants an urgent reassessment of the transmission dynamics of the current outbreak.

## Summary

Within family clusters that had SARS-CoV-2 infection, children had mild or even asymptomatic illness.

## Introduction

Over 1,600,000 cases of severe acute respiratory syndrome coronavirus 2 (SARS-CoV-2) infection have been confirmed globally and had resulted in up to 99,000 deaths by April 10, 2020. In early January 2020, the outbreak rapidly escalated, with hundreds of cases now confirmed within household clusters (1). Like influenza viruses, SARS-CoV-2 spreads primarily via respiratory droplets and causes infection by invading mucosa of the eyes, nose, or mouth (2). The underlying health status of the patient has been found to play a critical role in overall susceptibility in the SARS-CoV-2 outbreak (3). Indeed, novel coronavirus pneumonia infection within adult patients has been shown to cause rapid progression to acute respiratory distress syndrome (ARDS) and septic shock, commonly followed by multiple organ failure. According to the China Centers for Disease Control and Prevention (CDC) report, there have also been rare severe or fatal cases in SARS-CoV-2-infected children (4). In nine initial cases of hospitalized infants, the infants were vulnerable to the novel coronavirus but had mild illnesses (5). However, with a relatively small sample size and a limited pediatric age group, many critical issues for the formulation of measures to quarantine and treat children infected with SARS-CoV-2 still remain unclear. Therefore, in this research, a retrospective case study was conducted on 74 children in family clusters with confirmed COVID-19 infection in Wuhan Children's Hospital. The study provides the first delineation of the characteristics of children with COVID-19 infection in family clusters and meets the need to give much medical attention to COVID-19-infected children under the current viral crisis.

## Materials and Methods

### Study Design and Participants

A retrospective case study was performed in one hospital designated for children with COVID-19 infection, Wuhan Children's Hospital. This study was approved by the Ethics Committee of Wuhan Children's Hospital of Tongji Medical College, Huazhong University of Science and Technology. The requirement for informed patient consent was waived by the ethics committee due to it being a retrospective case study and the emergency nature of the COVID-19 infection outbreak. All the hospitalized COVID-9-infected children were identified between January 28, 2020, and March 3, 2020. The children diagnosed with COVID-19 through laboratory confirmation of SARS-CoV-2 infection by real-time polymerase chain reaction (RT-PCR) assay of nasal and pharyngeal swab specimens were enrolled in this study.

### Detecting SARS-CoV-2 by RT-PCR

During hospitalization, nasopharyngeal and anal swabs were collected and tested for SARS-CoV-2 RNA through reverse real-time polymerase chain reaction (RT-PCR) as described previously (6). The diagnostic criteria for COVID-19 were based on the recommendations in the Diagnosis and Treatment Protocol for the novel coronavirus pneumonia issued by the National Health Commission of the People's Republic of China. Infection was defined as the occurrence of at least one positive RT-PCR test result.

### Data Collection

Clinical data were retrospectively retrieved from the medical records. The date of disease onset was defined as the day when a symptom was first noticed. Information on the symptoms, laboratory, chest CT, and treatment during the hospital were also collected. ARDS was defined according to the Berlin definition (7).

### Statistical Analysis

For descriptive analysis, continuous variables were presented as mean ± SD or as median with Interquartile Range (IQR) whenever appropriate. Categorical variables were presented as a number and percentage The Shapiro-Wilk test was used to test the normality of data distribution. The Kaplan-Meier method was used on time-to-event data to estimate the median time and its corresponding 95% confidence interval (CI). All statistical analysis was performed using IBM SPSS Statistics for Windows version 26.0.

## Results

### The Demographic and Epidemic Characteristics of Children With COVID-19 Infection

The demographic and epidemic characteristics of the 74 COVID-19-infected children with confirmed SARS-CoV-2 infection are shown in Table 1. There were 38 male patients and 36 female patients, aged between 2 months and 15.3 years; the median age was 5.8 years, and 36 (48.7%) of the patients were over 6 years old, while 26 (35.1%) of the patients were younger than 3 years old. Fourteen (18.9%) of the children had a medical history of at least one previous condition (i.e., asthma, gastrointestinal ulcer, epilepsy, hepatolenticular degeneration, or acute lymphocytic leukemia). Seven (9.5%) of the COVID-19-infected children had previously been infected with other respiratory diseases.

**Table 1:** Characteristics of patients infected with SARS-CoV-2.

	**Total (*n* = 74%)**	**With symptoms (*n* = 52%)**	**Without symptoms (*n* = 22%)**	***P***
Gender				
Female	38 (51.4)	28 (53.8)	10 (45.5)	
Male	36 (48.6)	24 (46.2)	12 (54.5)	0.613
Age, median (IQR)	5.8 (1.1–9.8)	3.9 (0.9–10.1)	6.9 (4.7–9.8)	0.075
1m-	26 (35.1)	23 (44.2)	3 (13.6)	0.016
3y-^*^	12 (16.2)	5 (9.6)	7 (31.8)	0.034
6y−15y	36 (48.7)	24 (46.2)	12 (54.5)	0.616
Number of family members infected				
2	24 (32.4)	16 (30.8)	8 (36.4)	0.786
3	29 (39.2)	20 (38.5)	9 (40.9)	1.000
4	12 (16.2)	10 (19.2)	2 (9.1)	0.491
5	7 (9.5)	4 (7.7)	3 (13.6)	0.418
6	1 (1.4)	1 (1.9)	0	1.000
Medical history				
Respiratory	7 (9.5)	6 (11.5)	1 (4.5)	0.666
Digestive	2 (2.7)	2 (3.8)	0	1.000
Nervous	3 (4.1)	2 (3.8)	1 (4.5)	1.000
Metabolic system	1 (1.4)	1 (1.9)	0	1.000
Hematologic	1 (1.4)	0	1 (4.5)	1.000
CT				
Positive^*^	40 (54.1)	33 (63.5)	7 (31.8)	0.022
Single lobe	21 (28.4)	14 (26.9)	7 (31.8)	0.779
Multiple lobe^*^	19 (25.7)	19 (36.5)	0	0.001
Unilateral lung	26 (35.1)	18 (34.6)	8 (36.4)	1.000
Bilateral lung	15 (20.3)	15 (28.8)	0	0.003
GGO^*^	26 (35.1)	25 (48.1)	1 (4.5)	0.000
PCS	14 (18.9)	13 (24)	1 (4.5)	0.052
ILA	8 (10.8)	5 (9.6)	3 (13.6)	0.688
Negative^*^	34 (45.9)	19 (36.5)	15 (68.2)	0.021
Treatment				
Oxygen therapy	1 (1.4)	1 (1.9)	0	1.000
Glucocorticoid	1 (1.4)	1 (1.9)	0	1.000
Immunoglobulin	1 (1.4)	1 (1.9)	0	1.000
Oseltamivir	5 (6.8)	5 (9.6)	0	0.313
Inhaled interferon	74 (100)	52 (100)	22 (100)	

* *P < 0.05, with symptom vs. without symptom*.

*Data was shown as median (IQR)*.

Out of the 74 patients, two children came from the same family, while the others came from different families. Twenty-nine (39.2%) families had 3 patients, and 25 (33.8%) families had 2 patients. In these infected family members, in nearly 35 (46.7%) grandfathers and in 32 (42.7%) grandmothers were infected first in the family, while in only 21 (28.4%) were fathers and in 15 (20.3%) were mothers infected first. They were all infected before the children. The mean time between the first positive RT-PCR finding and the initial suspected child case in the family was 14.2 ± 6.1 days. Twenty-two of the 74 (29.7%) patients had no earlier symptoms, but they were hospitalized because they had had previous contact with infected relatives within their families or they were hospitalized for other reasons and were later diagnosed with COVID-19 through RT-PCR positive tests. In these asymptomatic patients, 3 of the 22 (13.6%) children were aged between 1 month to 3 years, 7 (31.8%) were aged between 3 and 6 years, and 12 (54.5%) were aged above 6 years.

### Clinic Features and Laboratory Findings of COVID-19 in Children

The clinic characteristics at admission time are listed in Table 1. The major clinical features of SARS-CoV-2 in children on initial presentation included: fever (51.4%), cough (55.4%), sputum (29.8%), and diarrhea (13.5%). Poor appetite, fatigue, vomiting, abdominal pain, and myalgia were present, but on rare occasions. In addition, 22 (29.7%) infected children had neither symptoms nor laboratory indications nor CT evidence for lesions. These asymptomatic patients only showed SARS-CoV-2 RNA positive. There were no differences between the children with symptoms and asymptomatic children in terms of median age, sex ratio, or the status of comorbidities (Table 1).

The laboratory indices and microbiologic findings on admission are shown in Table 2. No lymphopenia or thrombocytopenia was observed on admission in this study. Fourteen (18.9%) children demonstrated elevated levels of high-sensitivity C-reactive protein. The aspartate aminotransferase level on admission was elevated in 17 patients (23.0%), and the alanine aminotransferase level was elevated in three patients (4.1%). Kidney and heart function were regular in all cases. In the studied 74 patients, 28 (37.8%) were positive for mycoplasma, two were positive for EB-IgM, and one was positive for CMV-IgM. Of all of the nasopharyngeal aspirates collected, all patients were negative for influenza virus A and B. The differences in laboratory findings between the children with symptoms and asymptomatic children are listed in Table 2. Compared to patients without symptoms, the patients with symptoms showed higher levels of procalcitonin (PCT), lactate dehydrogenase (LDH), alanine aminotransferase (ALT), aspartate aminotransferase (AST), and creatinine (Cr).

**Table 2:** Laboratory tests of patients infected with 2019-nCoV on admission.

	**With symptom (*n* = 52) median (IQR)**	**Without symptom (*n* = 22) median (IQR)**	***P***
WBC (×10^9^/L, 3.85–10)	6.15 (5.28–8.77)	6.52 (5.47–7.54)	0.645
LYM (×10^9^/L, 1.15–4)	3.15 (1.98–4.66)	2.85 (1.87–3.79)	0.661
NEU (×10^9^/L, 1.08–5.8)	2.03 (1.67–3.40)	2.52 (1.87–3.79)	0.373
RBC (×10^12^/L, 4–5)	4.50 (4.27–4.73)	4.64 (4.33–4.82)	0.271
PLT (×10^9^/L, 100–320)	282.00 (236.00–363.00)	272.50 (232.50–348.50)	0.918
hsCRP (mg/L, 0–3)	0.25 (0.25–2.98)	0.25 (0.25–2.21)	0.418
PCT (ng/ml, ≤ 0.05)^*^	0.06 (0.04–0.08)	0.04 (0.04–0.06)	0.003
LDH (U/L, 175–322)^*^	267.00 (218.00–363.50)	225.00 (193.25–249.75)	0.005
CK (U/L, 30–170)	106.00 (70.00–151.00)	95.50 (63.75–134.50)	0.399
CK–MB (U/L, 0–25)	27.00 (20.50–40.50)	23.00 (18.50–32.75)	0.126
ALT (U/L, 15–46)^*^	19.00 (13.00–33.00)	12.00 (10.00–16.25)	0.003
AST (U/L, 21–72)^*^	38.00 (26.00–51.00)	24.00 (21.75–29.25)	0.000
γ-GT (U/L, 0–50)	12.00 (9.00–16.25)	11.00 (8.00–13.75)	0.207
BUN (mmol/L, 2.9–7.1)	4.12 (3.06–4.90)	4.37 (3.68–5.15)	0.259
Cr (μmol/L, 27–62)^*^	28.30 (23.30–40.60)	36.45 (31.35–43.03)	0.014
Co–infection, *n*			
EB-IgM (+)	2 (3.8%)	1 (4.5%)	1.000
CMV-IgM (+)	1 (1.9%)	1 (4.5%)	0.509
MP-IgM (+)	21 (40.4%)	7 (31.8%)	0.608

* *P < 0.05 with symptom vs. without symptom*.

*WBC, white blood cell; RBC, red blood cell; LYM, lymphocyte; NEU, neutrophil; PLT, platelet; hsCRP, high-sensitivity C-reactive protein; PCT, procalcitonin; LDH, lactate dehydrogenase; CK, creatine kinase; ALT, alanine aminotransferase; AST, aspartate aminotransferase; γ-GT, γ-glutamyltransferase; BUN, blood urea nitrogen; Cr, creatinine; EB, Epstein Barr virus; CMV, cytomegalovirus; MP, mycoplasma*.

### Radiological Features of COVID-19-Infected Children

**Figure 1:**
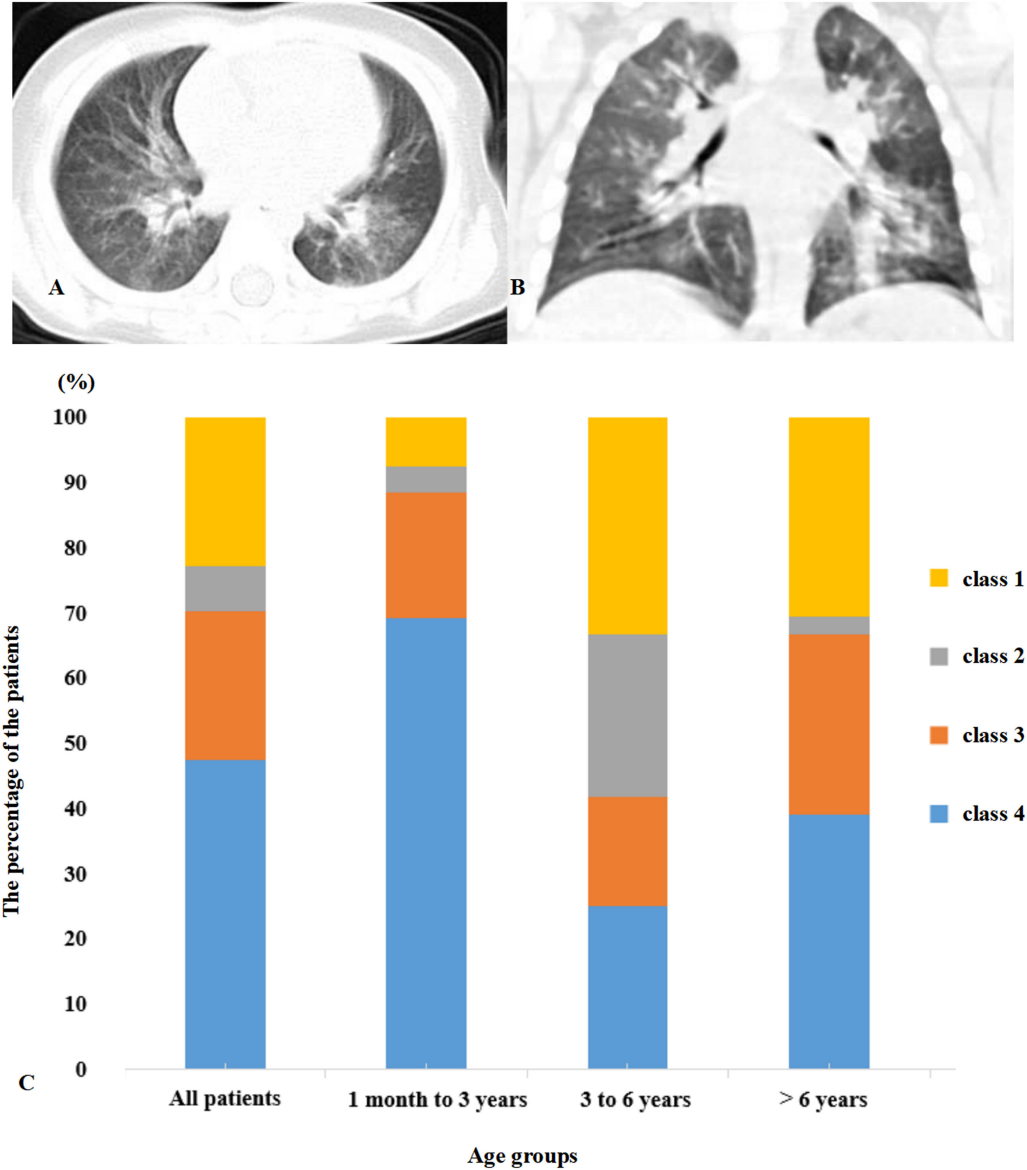
**(A,B)** Multifocal ground-glass opacities are shown in the lung. **(C)** Distribution of clinical symptoms and typical manifestations of viral pneumonia in chest CT. Class 1, symptom (+) CT (+); Class 2, symptom (+) CT (–); Class 3, symptom (–) CT (+); Class 4, symptom (–) CT (–).

Figures 1A,B display representative images of the chest CT from a child with COVID-19 Infection upon admission. Forty patients (54.1%) had abnormal findings in chest CT on admission. The CT images showed unilateral pulmonary infiltrate in 26 (35.1%) and bilateral pulmonary infiltrates in 14 (18.9%) of the COVID-19-infected children. The predominant manifestations of chest CT were ground-glass opacity (GGO), which was found in 26 (35.1%) children, patchy consolidation (found in 14, 18.9%), and interstitial abnormalities (found in 8, 10.8%). More positive findings were present in chest CT from children with symptoms in comparison to asymptomatic children (Table 1).

All of the children had at least one positive result in the RT-PCR test for viral RNA performed on nasopharyngeal swabs upon their admission to the hospital. Based on the respiratory and digestive symptoms and typical manifestations of viral pneumonia in chest CT, the children were divided into four groups (Figure 1C). Forty patients (54.1%) presented symptoms with typical positive CT results for viral pneumonia infection. Seventeen patients (23.0%) presented symptoms with an absence of the typical positive CT results for viral pneumonia infection, indicating upper respiratory tract infection or gastrointestinal infection of COVID-19. Five (6.7%) of the patients did not present any respiratory or digestive symptoms, but CT scan proved the presence of viral pneumonia infection. Seventeen (23%) children were classified as asymptomatic carriers with neither symptoms nor typical positive CT results for viral pneumonia infection.

### Treatment and Outcome

The infected children were under medical observation and quarantine. All of them received inhaled interferon with 2–4 μg/kg in 2 mL sterile water nebulization two times per day for 5–7 days.

By March 3, 2020, the body temperature returned to normal, and symptoms significantly improved in all of the infected children. The median time for release of fever was 3.0 (IQR of 1.0–4.0), and for all, the symptoms improved in 97.3% children with symptoms from the onset of the illness.

**Figure 2:**
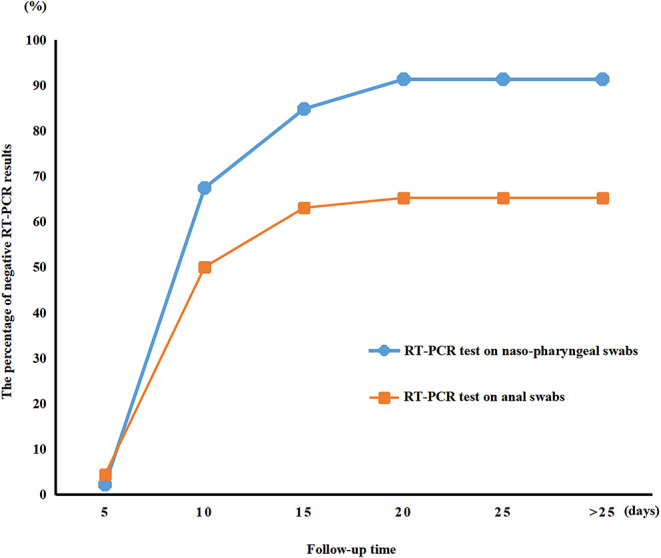
Percentages of negative nasopharyngeal swabs and anal swabs test during follow-up.

RT-PCR test results for viral RNA from anal swabs were available from 46 patients (62.2%). The anal swabs and nasopharyngeal swabs were taken and tested co-currently for each patient. Figure 2 shows the temporal pattern in the percentage of negative RT-PCR results for the nasopharyngeal or anal swabs of the 46 patients who had both their nasopharyngeal and anal swabs tested. The median time for nucleic acid tests to turn negative was 9.0 days (IQR of 7.0–13.0) for nasopharyngeal swabs and 10.0 days (IQR of 7.0–17.0) for anal swabs. The nasopharyngeal nucleic acid tests of 91.3% of children turned negative within 28 days from admission time. In comparison, fewer anal swab nucleic acid tests (65.2%) turned negative than did nasopharyngeal swab tests in 28 days (HR 1.718, 95% CI 1.040–2.839, *P* = 0.0346, Figure 1). Additionally, the median time for nucleic acid tests to turn negative was 9.0 days (IQR of 7.0–13.0) for nasopharyngeal swabs and 10.0 days (IQR of 7.0–17.0) for anal swabs.

## Discussion

We described the epidemiological and clinical characteristics of 74 children patients with confirmed COVID-19 infection by family cluster. Most children with COVID-19 infection were toddlers and schoolchildren without any gender susceptibility. The average internal time between the diagnosis of the children with a positive nucleic acid test and the initial case in their families was 14.2 ± 6.1 days. In most cases, it was a grandparent that was infected before the child. Cough, fever, and sputum occurred in most of the patients. Compared to nasopharyngeal swab, which turned negative after a median time of 9.0 (IQR of 7.0–13.0) days, it took a median time of 10.0 (IQR of 7.0–17.0) days for anal swab to turn negative. These mismatched results indicate that the body took longer to clear the virus from the digestive tract than from the respiratory tract. Regular lymphocytes and neutrophils, normal kidney and heart function, fewer severe cases, and a lower fatality rate were observed in the children. Apart from the typical CT images, half of the negative CT scans were from children with COVID-19 infection that presented some clinical symptoms. Furthermore, 29.7% of the children were asymptomatic carriers with positive SARS-CoV-2 nucleic acid testing results due to previous exposure to COVID-19-infected family members. This indicated that asymptomatic children with COVID-19 infection can be significant transmitters of the virus and can increase the number of cases of infection.

Since the large-scale outbreak of the COVID-19 virus infection in Wuhan, cases in the initial clusters reported that wild animals were the likely source, and an animal-to-human route was probably the main mode of transmission for those initially reported cases (8). Recently, nine SARS-CoV-2-infected infants were identified by Dr. Zhang and it was found that family clustering occurred for all of the infected infants (9). Similarly, in our cohort, COVID-19 in all of the 74 children was attributed to the family cluster (10). SARS-CoV-2 was transmitted to these children by infected adult relatives such as parents or grandparents, by close exposure. Incredibly, one family had seven COVID-19 patients. They were passively screened for SARS-CoV-2 nucleic acid due to exposure to an infected relative or sought medical treatment for other reasons. Just as the transmission pattern of MERS-CoV involves transmission within families (11), SARS-CoV-2 was also transmitted in family clusters. In our present study, all of the child patients had been infected by a family member. It was mostly a grandfather or grandmother that was infected first in the family. These data indicated that elderly relatives were still an important source of infection of SARS-CoV-2 in family clusters in China. Traditionally, family groups in China are large, with children living with their parents and grandparents. However, Niccolò et al. reported that 55% of child patients were exposed to SARS-CoV-2 from unknown sources or from sources outside the child's family in Italy (12). The longest interval between the diagnosis of a child via positive nucleic acid test and the initial case in his or her family was over 1 month (42 days). Furthermore, 29.7% of children had no symptoms or signs, which might lead to a delay in medical treatment, which might cause transmission to more people.

In the present study, most of the COVID-19-infected children were younger than 3 years and older than 6 years. Similar to SARS and MERS cases, there is a lot of variability in the clinical presentation, including mild or asymptomatic cases that may never be presented to healthcare services (4, 13), Compared with adult cases, most of the children with COVID-19 exhibited mild or moderate symptoms. The main clinic signs of COVID-19 in children are fever and cough. However, child patients manifested sputum instead of the adult dry cough (14) (Table 1). In addition, a few child patients suffered from diarrhea, which was uncommon in adults. Diarrhea was also reported to be associated with Middle East Respiratory Syndrome coronary virus (MERS-CoV) infection (15, 16). This suggests the possibility of direct viral involvement of the alimentary canals. Compared to adults with COVID-19, in whom the virus mainly acted to reduce lymphocytes, none of the children with COVID-19 had lymphopenia (Table 2). These might indicate a lack of an over-activated immune response in children with COVID-19. In contrast, adults infected by SARS-CoV-2, severe acute respiratory syndrome coronavirus (SARS-CoV), or MERS-CoV were reported to have increased concentrations of proinflammatory cytokines, which were associated with pulmonary inflammation and extensive lung damage in patients (17, 18). This difference might explain the mild symptoms in children with COVID-19. Recently, Toubiana reported that SARS-CoV-2 infected children and adolescents might related to the ongoing outbreak of Kawasaki-like multisystem inflammatory syndrome among children and adolescents in the Paris area. However, we did not find that the patients manifested the same clinical signs as Kawasaki disease (19). Children are susceptible to general respiratory diseases. In our present study, none of the COVID-19-positive children were co-infected with other bacteria or A/B flu. While few patients were co-infected with cytomegalovirus or EB virus, 37.8% of the children with COVID-19 were co-infected with mycoplasma (MP), especially patients over 6 years old. MP pneumoniae is one of the most common causes of childhood community-acquired pneumonia (9). Our data showed MP IgM positive results, which indicates recent infection with MP. It is not possible to determine which infected came first, MP or SARS-CoV-2 or whether MP-infected children might be more susceptible to SARS-CoV-2.

Of the 74 COVID-19-infected children, 54.1% of patients had positive CT images, which was lower than for COVID-19-infected adult patients, in whom there are 75% positive chest CT findings (20). Compared to the group without symptoms, more patients showed positive CT in the group with symptoms. This indicated that lung injury accompanied the symptoms in children. Typical CT manifestations of the viral pneumonia are mainly multifocal ground-glass changes. The manifestations of lung CT scans in adult patients were bilateral, subpleural, ground-glass opacities with air bronchograms, ill-defined margins, and a slight predominance in the right lower lobe (21). Partly similar to adult patients lung injure entexisted in unilateral lung and a single lung lobe in child patients. In adults, COVID-19 pneumonia manifests with chest CT imaging abnormalities, even in asymptomatic patients infected with SARS-CoV-2. However, 23.0% of COVID-19-positive children were classified as asymptomatic carriers who had neither symptoms nor radiological findings. This accords with a publication that reported that 20.9% of adult patients have isolated SARS-CoV-2 infection before or without the development of viral pneumonia. However, CT might not always be necessary but could be performed upon clinical suspicion, and lung ultrasound can be a useful tool (22, 23). Furthermore, there were significant differences in laboratory results between the asymptomatic group and symptomatic group. Compared to the patients without symptoms, there were higher levels of LDH, ALT, AST, and Cr in the patients with symptoms. These data indicate that the patients suffered mild organ damage, particularly of the heart, liver, and kidney. Most child cases missed by screening are fundamentally undetectable because they have not yet developed symptoms and are unaware that they were exposed (14). These findings indicate that not all children with COVID-19 suffer lung damage and also advocate for shifting the focus in SARS-CoV-2 screening to children once a family member is confirmed to have COVID-19.

SARS-CoV-2-positive detection from nasopharyngeal swabs by PCR is the basis for diagnosis of COVID-19 in our present study. In contrast to negative results from nasopharyngeal swab, 34% of anal swabs continued to show positive. Simultaneously, diarrhea was also presented in children COVID-19. Studies have demonstrated that patients with a positive stool test did not experience gastrointestinal and respiratory symptoms (24). SARS-CoV-2 infection through transmission through stool should then be considered, and a fecal-oral route could be a potential transmission pathway. Therefore, we should strengthen stool management and be alert to fecal-oral spread when caring for children.

The study indicates that asymptomatic children within a family cluster are potentially a little-known source of COVID-19, and this warrants an urgent reassessment of the transmission dynamics of the current outbreak. Moreover, pediatric experts have suggested that imaging should not be used routinely for child patients with COVID-19 who are asymptomatic or have mild symptoms (22, 25). To really understand the real burden of pediatric COVID-19, particularly in children exposed to adults with COVID-19, serologic studies of these children might be important (26). It is also recommended that both nasopharyngeal and anal swabs should be confirmed negative as a standard for release. Such a situation makes the control and management efforts for the spread and transmission of the viral infection much more challenging, hence increasing the chances of infection via transmission, a situation that could be the reason for the spread of COVID-19 infection to other parts of the world. Therefore, there is still a need for much effort on early identification and timely treatment, especially of asymptomatic cases in children, as this is crucial in fighting the pandemic, not only in China but the world over.

## Data Availability Statement

The raw data supporting the conclusions of this article will be made available by the authors, without undue reservation.

## Ethics Statement

The studies involving human participants were reviewed and approved by Ethics Committee of Wuhan Children's Hospital of Tongji Medical College of Huazhong University of Science and Technology. Written informed consent to participate in this study was provided by the participants' legal guardian/next of kin. Written informed consent was obtained from the individual(s), and minor(s)' legal guardian/next of kin, for the publication of any potentially identifiable images or data included in this article.

## Author Contributions

This study was designed, directed, and coordinated by DS, HP, FZ, and JW. XC, HX, ZW, XL, and JM collected the epidemiological and clinical data. ZL and HX collected the CT images and designed the figures. CW and JL performed the analytical calculations. DS, HP, and FZ drafted the manuscript. JW, JM, and ZL revised the final manuscript. All authors approved the final manuscript as submitted and agree to be accountable for all aspects of the work.

## Conflict of Interest

The authors declare that the research was conducted in the absence of any commercial or financial relationships that could be construed as a potential conflict of interest.

## Abbreviations

COVID-19: Coronavirus infection of 2019
SARS-CoV-2: severe acute respiratory syndrome coronavirus 2
ARDS: acute respiratory distress syndrome
RT-PCR: reverse real-time polymerase chain reaction
IQR: Interquartile Range
CI: confidence interval
GGO: ground-glass opacity
SARS-CoV: severe acute respiratory syndrome coronavirus
MERS-CoV: Middle East Respiratory Syndrome coronary virus.
